# Changes in calcitonin gene-related peptide (CGRP) receptor component and nitric oxide receptor (sGC) immunoreactivity in rat trigeminal ganglion following glyceroltrinitrate pretreatment

**DOI:** 10.1186/1129-2377-14-74

**Published:** 2013-09-03

**Authors:** Kristin Seiler, Judith I Nusser, Jochen K Lennerz, Winfried L Neuhuber, Karl Messlinger

**Affiliations:** 1 Institute of Physiology & Pathophysiology, University of Erlangen-Nürnberg, 91054 Erlangen, Germany; 2 University Ulm, Institute of Pathology, Ulm, Germany; 3 Institute of Anatomy, University of Erlangen-Nürnberg, Erlangen, Germany

**Keywords:** CGRP receptor, sGC, Nitroglycerin, Trigeminal ganglion, Headache

## Abstract

**Background:**

Nitric oxide (NO) is thought to play an important role in the pathophysiology of migraine. Infusion of the nitrovasodilator glyceroltrinitrate (nitroglycerin, GTN), which mobilizes NO in the organism, is an approved migraine model in humans. Calcitonin gene-related peptide (CGRP) is regarded as another key mediator in migraine. Increased plasma levels of CGRP have been found during spontaneous as well as nitrovasodilator-induced migraine attacks. The nociceptive processes and interactions underlying the NO and CGRP mediated headache are poorly known but can be examined in animal experiments. In the present study we examined changes in immunofluorescence of CGRP receptor components (CLR and RAMP1) and soluble guanylyl cyclase (sGC), the intracellular receptor for NO, in rat trigeminal ganglia after pretreatment with GTN.

**Methods:**

Isoflurane anaesthetised rats were intravenously infused with GTN (1 mg/kg) or saline for four hours and two hours later the trigeminal ganglia were processed for immunohistochemistry. Different primary antibodies recognizing CLR, RAMP1, CGRP and sGC coupled to fluorescent secondary antibodies were used to examine immunoreactive cells in serial sections of trigeminal ganglia with epifluorescence and confocal laser scanning microscopy. Several staining protocols were examined to yield optimized immunolabeling.

**Results:**

In vehicle-treated animals, 42% of the trigeminal ganglion neurons were immunopositive for RAMP1 and 41% for CLR. After GTN pretreatment CLR-immunopositivity was unchanged, while there was an increase in RAMP1-immunopositive neurons to 46%. RAMP1 and CLR immunoreactivity was also detected in satellite cells. Neurons immunoreactive for sGC were on average smaller than sGC-immunonegative neurons. The percentage of sGC-immunopositive neurons (51% after vehicle) was decreased after GTN infusion (48%).

**Conclusions:**

Prolonged infusion of GTN caused increased fractions of RAMP1- and decreased fractions of sGC-immunopositive neurons in the trigeminal ganglion. The observed alterations are likely immunophenotypic correlates of the pathophysiological processes underlying nitrovasodilator-induced migraine attacks and indicate that signalling via CGRP receptors but not sGC-mediated mechanisms may be enhanced through endogenous NO production.

## Background

Calcitonin gene related peptide (CGRP), which is expressed in a major part of primary afferent neurons, is known to play an important role in migraine and other primary headaches [1-3]. Increased plasma levels of CGRP have been found in the jugular vein of migraineurs during spontaneous and nitrovasodilator-induced migraine attacks [4-6]. Infusion of CGRP induced headaches in migraineurs, partly fulfilling diagnostic criteria of migraine, but less frequently in healthy persons [7,8]. Headaches induced by CGRP infusion in healthy test persons were prevented by the CGRP receptor antagonist olcegepant (BIBN4096BS) [8]. Olcegepant and the later developed orally available CGRP receptor antagonist telcagepant (MK-0974) proved to be effective in the treatment of spontaneous migraine attacks in well-designed clinical phase III studies [9,10].

In animal experiments topically applied CGRP receptor antagonists (CGRP_8-37_ and olcegepant) inhibited increases in meningeal blood flow elicited by periodic electrical field stimulation of rat dura mater [11,12]. Spontaneous, heat-evoked and nitrovasodilator-induced activity of rat spinal trigeminal neurons with meningeal afferent input was reduced when CGRP receptor antagonists were intravenously infused but local administration of olcegepant onto meningeal receptive fields was ineffective [13,14]. However, the activity of neurons in the upper cervical dorsal horn with meningeal afferent input was modulated by microiontophoretic application of CGRP and CGRP receptor antagonists, which indicates a role for central CGRP receptors in the control of spinal trigeminal activity [15].

The CGRP receptor consists of a large peptide with seven transmembrane domains, the calcitonin receptor-like receptor (CLR or CRLR) complemented by a single transmembrane domain, the receptor activity-modifying protein 1 (RAMP1). RAMP1 is responsible for the CGRP specificity, whereas the association of CLR with other receptor activity-modifying proteins like RAMP2 or RAMP3 commutes the receptor to an adrenomedullin receptor [16]. For the constitution of the functional CGRP receptor a third intracellular component, the receptor component protein (RCP), which couples the receptor to the intracellular signal pathway through Gs proteins and adenylyl cyclase, is essential [17]. CGRP receptors are widely expressed in the central nervous system, in sensory ganglia and in blood vessels [18]. In the trigeminal ganglion CGRP receptor immunoreactivity has been found in neurons and satellite glial cells [19-21].

Various studies indicate that NO plays also an essential role in the pathogenesis of migraine [22,23]. Infusion of the nitrovasodilator glycerol trinitrate (GTN, 0.5 μg/kg over 20 min) into migraineurs and healthy control persons caused immediate and delayed headaches. Delayed headaches, which were typically experienced by migraineurs peaking on average four hours after the infusion, were more severe and fulfilled the diagnostic criteria for migraine in half of the migraine group [24].

The links from NO to the pathophysiological processes in migraine are not yet resolved. Soluble guanylyl cyclase (sGC), a heterodimer consisting of an alpha and a beta subunit [25,26], acts as the intracellular receptor for NO and catalyses the conversion of GTP into the second messenger 3′,5′-cyclic guanosine monophosphate (cGMP) [27]. In rat dural blood vessels increased sGC expression and activity have been observed after subcutaneous injection of 10 mg/kg GTN [28]. After pretreatment of rats with GTN (250 μg/kg) the proportion of trigeminal ganglion neurons immunoreactive for CGRP as well as for neuronal NO synthase (nNOS) was increased [29]. Provided that this reflects an increase in CGRP and NO production, it may result in elevated release of CGRP and NO from trigeminal afferents and could contribute to the delayed activation of rat spinal trigeminal neurons observed after nitrovasodilator administration [14,30].

In the present study we asked if pretreatment with an NO donor changes the equipment of trigeminal neurons with receptor components of the CGRP and NO signalling systems detected by immunofluorescence. The results provide immunophenotypic evidence for a regulation of RAMP1 and sGC at the level of the trigeminal ganglion.

## Methods

### Animals

Fourteen adult male Wistar rats (250–400 g) were used for the study (Charles River, Wilmington, MA). Rats were bred and kept on a 12 hour light 12 hour dark cycle with unlimited access to water and food. The University of Erlangen-Nürnberg and the local government approved the experimental protocols. All procedures were conducted in accordance with the European Communities Council directive of animal care and the German regulations of animal welfare and treatment, and the experimental protocols were reviewed by the local district government.

### Preparation and tissue processing

Fourteen laboratory animals were initially anaesthetised in a closed box by inhaling 4% isoflurane (Abbott, Wiesbaden, Germany). Anaesthesia was maintained for six hours by spontaneous breathing of humidified and oxygen-enriched (30%) air with 2% isoflurane through a loosely fitted mask. Rats were placed in prone position on a feedback-controlled warming plate to maintain the body temperature of 37°C. A catheter was introduced into the femoral vein to administer substances diluted in isotonic saline by a syringe pump (Harvard Apparatus, March-Hugstetten, Germany). The NO donor glyceroltrinitrate (GTN, Schwarz Pharma, Monheim, Germany) was continuously applied intravenously (i.v.) at a dose of 1 mg/kg bodyweight over four hours. Control animals obtained isotonic saline as vehicle over an identical period and with an identical infusion rate. All rats were kept anaesthetised for another period of two hours receiving isotonic saline at an infusion rate of 0.5 ml/h before being euthanized with an overdose of Trapanal (Thiopental, Nycomed, Konstanz, Germany) and perfused. Two rats were directly perfused after anaesthesia without any infusions in order to control possible effects of the anaesthetic procedures.

After thoracotomy, warm isotonic saline was perfused through the left ventricle for about 3 minutes, in some of the experiments followed by fixative (see below). The head was separated from the body, divided in the sagittal plane and the brain was removed. Both trigeminal ganglia were freed from the wrapping dura mater and carefully excised from the skull base. In those cases, in which the ganglia remained unfixed for staining, they were mounted on a base of frozen Tissue-Tek (Slee, Mainz, Germany) on small plastic forms, then rapidly frozen in liquid nitrogen and stored in the freezer at −20°C. The ganglia were cut into series of 20 μm thick longitudinal sections using a cryostat (Leica, Bensheim, Germany). Sections were mounted on alternated poly-L-lysine-coated slides (Sigma-Aldrich, Steinheim, Germany) and dried for one hour at room temperature before staining.

**Figure 1:**
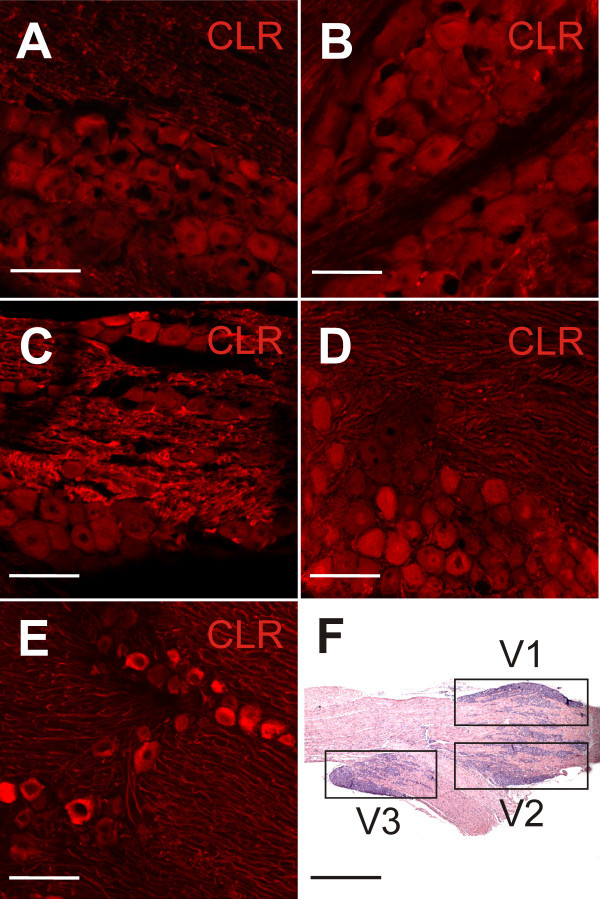
**Confocal images of trigeminal ganglion sections treated with the rabbit anti-rat CLR antibody and the secondary goat anti-rabbit antibody conjugated to Cy3 after different fixation methods. (A)** Short Zamboni perfusion ~5 min, post-fixed in Zamboni for two hours. **(B)** Perfusion with 4% paraformaldehyde (PFA) for 20 min and postfixation in 4% PFA (2 h). **(C)** Perfusion with 4% PFA for ~5 min, postfixation in Zamboni (2 h). **(D)** Cryofixation, postfixation with methanol-acetone (7:3) for 10 min. **(E)** Cryofixation without any postfixation. **(F)** Low-power micrograph of a trigeminal ganglion section, stained with azur methylene blue, with the ophthalmic (V1), maxillary (V2) and mandibular division (V3), in which separate cell counts were made. Scale bars A-E = 100 μm; F = 1 mm.

In order to optimize immunostaining we compared the results of different fixation methods using the rabbit anti-rat CLR antibody (see below). All fixation procedures resulted in similar proportions of CLR-immunopositive trigeminal ganglion neurons but both the contrast and regularity of immunostaining showed some differences (Figure 1A-E). The first fixation test group of rats was perfused after saline with Zamboni fixative (solution of 0.01% picric acid and 4% paraformaldehyde (PFA) in 10 mM phosphate-buffered saline (PBS), pH 7.4) for 5 min. The removed ganglia were post-fixed in Zamboni for two hours, stored in 10 mM PBS for 24 hours and kept in a 30% solution of sucrose in PBS for cryoprotection before they were cut and immunostained at the next day (Figure 1A). The second test group was perfused with a solution of 4% PFA in 10 mM (PBS, pH 7.4) for 20 minutes, postfixed in the same fixation solution for two hours and stored in 10 mM PBS for 24 hours. Cryoprotection was accomplished in a 30% solution of sucrose in PBS for one day (Figure 1B). The third test group was perfused with a solution of 4% PFA in PBS for ~5 minutes, postfixed in Zamboni (2 h), stored in 10 mM PBS for 24 hours and put in a 30% solution of sucrose in PBS for cryoprotection for one day (Figure 1C). The fourth test group of animals was perfused with isotonic saline for about three minutes, the removed trigeminal ganglia were rapidly frozen in liquid nitrogen and sections were postfixed on slides in a mixture of methanol and acetone (7:3) for ten minutes directly before staining (Figure 1D).

Unfixed cryopreserved trigeminal ganglia showed the most consistent quality and the best contrast of stained structures. Therefore, this method was used for quantification of neurons (Figure 1E).

### Immunohistochemistry

The sources, characteristics, and dilutions of the primary and secondary antibodies used in this study are listed in Tables 1 and 2. After rinsing in phosphate-buffered saline (PBS; 0.01 M, pH 7.4), the mounted sections were preincubated for one hour at room temperature with a solution of 5% donkey or goat serum (Dianova, Hamburg, Germany), containing PBS/BSA/TX; composed of PBS, 0.5% Triton X-100 and 1% bovine serum albumin. Sections were rinsed in PBS for another five minutes before incubating them with primary antibodies raised in rabbit or goat and directed against CLR, RAMP1, sGC ß1, or CGRP at room temperature overnight (characteristics and dilutions see Table 1). Sections were washed with PBS three times for five minutes and incubated with fluorescent secondary antibodies for 1 hour at room temperature. Secondary antibodies were goat anti-rabbit IgG coupled to indocarbocyanine (Cy3), donkey anti-goat IgG coupled to Alexa Fluor 488, donkey anti-rabbit IgG coupled to Alexa 555 (see Table 2). Sections were rinsed again three times for five minutes with PBS, coverslipped in Fluoromount G (SouthernBiotech, Birmingham, AL), and stored at 4°C. In selected immunostainings DAPI (4′,6′-diamidino-2-phenylindole hydrochloride; Sigma-Aldrich, St. Louis) for labelling of nuclear DNA was added at a concentration of 2 μg/ml to the secondary antibody.

**Table 1:** List of primary antibodies (all diluted in PBS/BSA/TX)

**Antigen**	**Host**	**Antigen characteristics/epitope**	**Reference number**	**Producer**	**Dilution**
Calcitonin receptor-like receptor (CLR)	Rabbit	GYSHDCPTEHLNGK in the C-terminal of human CLR	3152	Merck & Co., Inc. Rahway NJ, USA	1:1500
Calcitonin receptor-like receptor (CLR)	Rabbit	C-terminal of rat CLR	3155	Merck & Co., Inc. Rahway NJ, USA	1:500
Receptor activity modifying protein (RAMP1)	Goat	C-terminal of human RAMP1	844	Merck & Co., Inc. Rahway NJ, USA	1:100
Receptor activity modifying protein (RAMP1)	Rabbit	C-terminal of rat RAMP1	3158	Merck & Co., Inc. Rahway NJ, USA	1:2500
Soluble guanylyl cyclase (sGC ß1) (C-13)	Goat	Carboxy terminus of human sGC ß1	sc-21310	Santa Cruz Biotechnology, Santa Cruz, CA, USA	1:100
Calcitonin gene-related peptide (CGRP)	Goat	Rat Tyr-CGRP (23–27)	BT 17-2090-07	Biotrend, Cologne, Germany	1:100

**Table 2:** List of secondary antibodies (all diluted in PBS/BSA/TX)

**Secondary antibody**	**Conjugated to**	**Producer**	**Dilution**
Donkey anti-goat	Alexa 488	Molecular probes,	1:2000
		Eugene, OR,	
		USA	
Goat anti-rabbit	Cy3	Dianova,	1:200
		Hamburg,	
		Germany	
Donkey anti-rabbit	Alexa 555	Invitrogen,	1:1000
		Carlsbad, CA,	
		USA	

### Controls

To verify the specificity of the immunohistochemical reactions, every staining was controlled by omitting primary antibodies in the first incubation fluid, and the immunostaining was only accepted when these negative controls did not show any specific staining. Preabsorption controls have been accomplished for the CLR and RAMP1 primary antibodies in prior studies and there was no staining with preabsorbed CLR or RAMP1 antibodies using their respective blocking peptides [19]. In case of the goat anti-sGC ß1 antibody, the preabsorption control showed no specific staining (sGC peptide: sGC primary antibody 10:1). The anti-CGRP primary antibody used was evaluated by an enzyme-linked immunosorbent assay (ELISA; manufacturer’s technical information) and produced a pattern of CGRP-ir that was identical to previous descriptions [29,31-33]. In addition, evidence for the specificity of the goat anti-CGRP primary antibody resulted from virtually identical stainings as reported with other CGRP antibodies [34-36].

### Cell counting

For a quantitative analysis of neurons immunopositive for the relevant antigen, five longitudinal sections adjacent to the center of each ganglion showing all three trigeminal divisions, V1 (ophthalmic), V2 (maxillary) and V3 (mandibular), were selected for counting. Sections were taken from both trigeminal ganglia. To avoid double counting of neurons, every second section was omitted. The V1, V2 and V3 regions were analysed as a whole and also separately (see Figure 1F). All sections showed a faint background staining, which served to count the whole cell number. At least 33 neurons per anatomical region were counted, i.e. more than 500 neurons per ganglion. CLR, RAMP1, sGC ß1, CGRP, as well as double-stained neurons in various combinations were counted by the investigators, who were blinded to the treatment of rats using a 20× dry objective lens. In a sample of 10 sections both investigators (JN and KS) counted CLR-immunopositive and -negative neurons independently from each other and came to similar results (KS: 29% positive cells, total cell count 2411; JN: 32% positive cells, total cell count 2433). For the main experiments neurons were counted concordantly by both investigators in same session.

### Confocal microscopy and image processing

Sections were examined and images were obtained using a LSM 780 confocal-laser scanning system (Carl Zeiss MicroImaging GmbH, Jena, Germany) equipped with an Argon laser (458, 488, 514 nm), a diode laser (405 nm), a DPSS-laser (561 nm) and a HeNe-Laser (633 nm) (LASOS Lasertechnik, Jena, Germany), mounted on an inverted Axio Observer Z1. The filter settings of the confocal scanner for single and double labeling were: 488 nm excitation for Alexa 488 (beam splitter MBS 488, gritfilter 493–630 nm), 514 nm excitation for Cy3 (beam splitter MBS 458/514, gritfilter 538–681 nm) and Alexa 555 (beam splitter MBS 458/514, gritfilter 545–697 nm). Two dry objective lenses, (10× and 20× with numerical apertures of 0.3 and 0.8), two oil-immersion objective lenses (20× and 63× with numerical apertures of 0.8 and 1.4), and a 40× water objective lens (numerical aperture 1.3) were used. Electronic zoom factors varied between 1.0 and 1.4*.* Sequential scanning and appropriate pinhole settings were used to minimize spectral bleed through. For examination of co-localization of immunofluorescence, single optical sections at the same focus plane were taken separately and the two corresponding channels were merged. Adjustment for contrast, brightness and evenness of illumination was performed and exclusive areas of images were electronically enlarged in order to document details. The number of image pixels varied between 2048 × 2048 and 512 × 512 pixels. Channels of each picture were merged into a 12-bit RGB tiff-file using confocal assistant software ZEN 2010. For organising the final layouts and applying text and scale bars CorelDraw (Corel, Dublin, Ireland) was used.

### Statistical analysis

The percentage rate of neurons positive for CLR, RAMP1, sGC ß1, CGRP, as well as co-localized CLR/RAMP1 and sGC ß1/CGRP immunoreactivity was compared between GTN and saline-treated animals. In addition we separately analysed the distribution of cells immunopositive for these proteins in medial (ophthalmic, V1) and lateral (maxillary, V2 and mandibular, V3) regions of the trigeminal ganglion. Statistical analysis was performed with Statistica software (Tulsa, OK, USA). The nonparametric Chi-square test for independent samples was used to compare the number of immunopositive neurons between pretreatments. Significance was defined as p < 0.05. Data are reported as mean ± standard deviation (SD).

## Results

### Comparison of CLR and RAMP1 immunostaining using different antibodies

**Figure 2:**
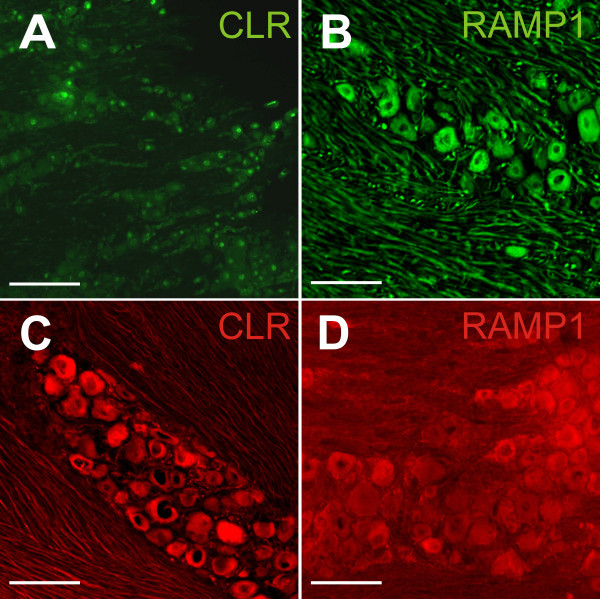
**Comparison of the CLR and RAMP1 immunofluorescence in trigeminal ganglia using different antibodies. (A)** Rabbit anti-human CLR antibody coupled to Alexa 488 (green) shows partly nuclear staining while neuronal cell bodies lack immunofluorescence. **(B)** Anti-human RAMP1 antibody coupled to Alexa 488 (green) produced homogenous or granulated staining of neuronal cell plasma but no nucleus staining. This antibody was used for the main experiments with cell counting. **(C)** Rabbit anti-rat CLR antibody coupled to Cy3 (red), resulting in mostly homogenous staining. It was finally used for the experiments with cell counting. **(D)** Rabbit anti-rat RAMP1 coupled to Cy3 (red) caused weak and less specific staining of neurons. Scale bars = 100 μm.

**Figure 3:**
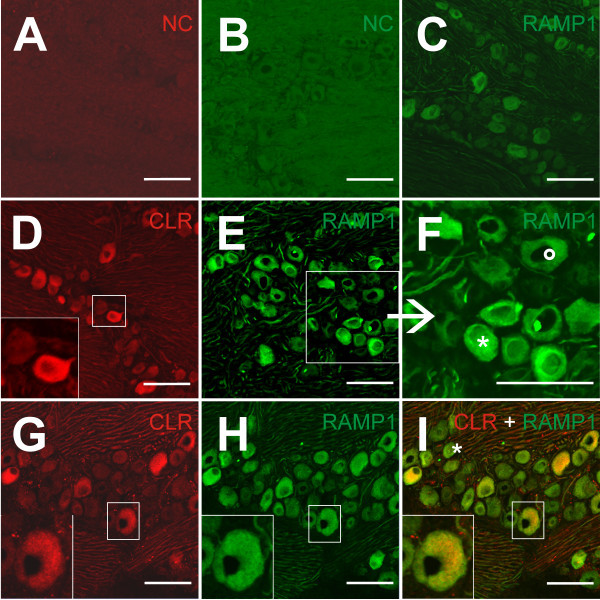
**CLR and RAMP1 immunostaining of trigeminal ganglia. (A)** CLR- and **(B)** RAMP1-negative controls (NC); primary antibodies were omitted and no specific immunostaining is visible after incubation with the secondary antibodies coupled with Cy3 **(A)** or Alexa 488 **(B)**. **(C)** RAMP1 immunostaining (Alexa 488) without any pretreatment of the animals. **(D)** CLR coupled to Cy3 (red) (saline-treated animal) and **(E)** RAMP1 coupled to Alexa 488 (green) (GTN-treated animal) immunofluorescence in the trigeminal ganglion. The magnified inset in **(D)** shows a typical CLR-immunopositive- and a CLR-immunonegative neuron. The magnified part of **(E)** in **(F)** illustrates the different intensity of the RAMP1 immunostaining of neurons. The neuron marked with * is a very intense RAMP1 immunopositive neuron compared to the neuron marked with ° but both were counted as immunopositive. **(G-I)** Double immunostaining using CLR and RAMP1 antibodies (GTN-treated animal). **(G)** CLR-positive neurons in the red channel, **(H)** RAMP1-positive neurons in the green channel and **(I)** both receptor components in the merged image; CLR and RAMP1 were colocalized in some neurons (yellow). Neurons exclusively immunopositive for CLR are extremly rare, in contrast to only RAMP1-positive neurons (*). The magnification shows a neuron immunopositive for both receptor components. All images from cryofixed trigeminal ganglia without any postfixation. Scale bars = 100 μm.

Antibodies against human (CLR 3152 and RAMP1 844) and rat (CLR 3155 and RAMP1 8158) CGRP receptor components were tested (Figure 2A-D). Generally, in neurons antibodies showed homogeneous or faintly granulated immunofluorescence. The anti-human CLR 3152 antibody coupled to Alexa 488 revealed some nuclear staining while the neuronal cell bodies lacked fluorescence (Figure 2A). The anti rat CLR 3155 antibody coupled with Cy3 stained the whole cytoplasm without any nucleus staining (Figure 2C). The staining was constant and evenly distributed throughout the whole ganglia. Therefore this antibody was selected for quantitative analysis (see Figure 3D). The anti rat RAMP1 3158 antibody coupled to Cy3 showed ambiguous staining, therefore a reliable distinction between positive and negative neurons was not always possible (Figure 2D). In contrast, the anti human RAMP1 844 antibody coupled to Alexa 488 showed clear and constant neuronal staining throughout the whole ganglia (Figure 2B); some neurons exhibited very intense fluorescence, while others were less intensely stained; both were evaluated as immunopositive. This RAMP1 antibody was selected for quantitative receptor analysis (see Figure 3E), because it showed most frequently a consistent quality of reliable staining, and in addition, due to species distinctions, double labelling with the rabbit anti-rat CLR 3155 antibody was easily possible. The anti human RAMP1 844 antibody has previously been demonstrated to work well in rat tissue [19].

### Proportion of CLR and RAMP1-immunoreactive neurons

**Figure 4:**
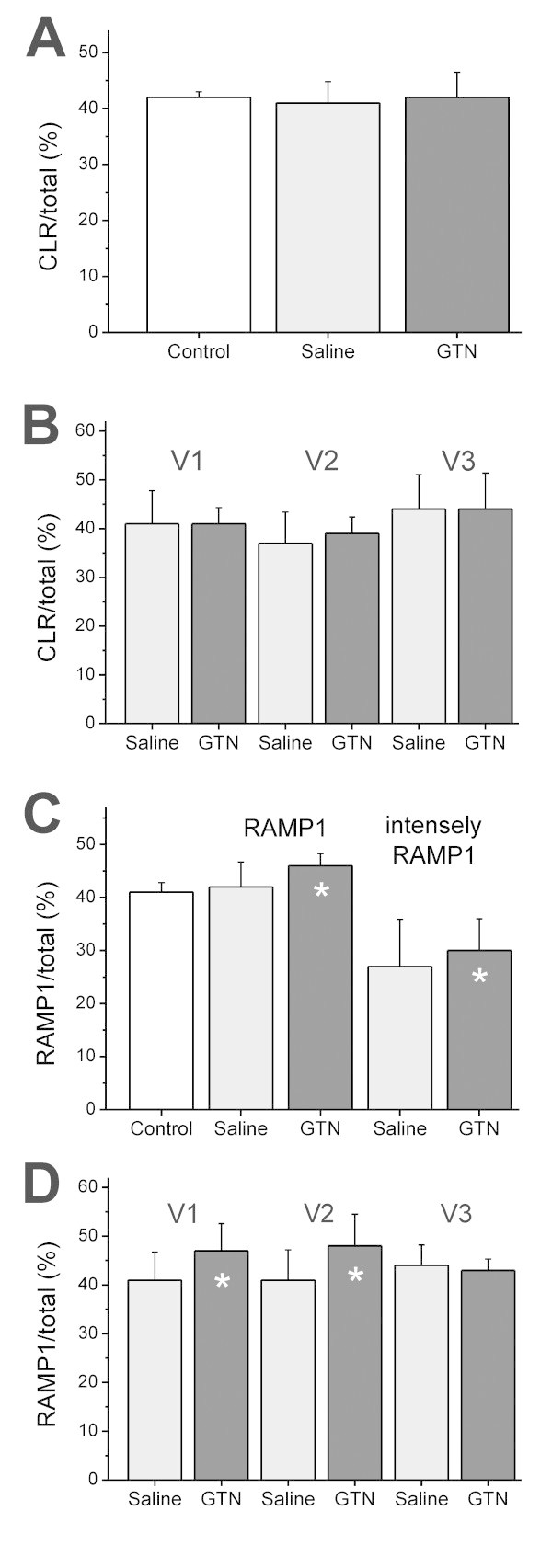
**Quantitative analysis of CLR- and RAMP1-immunopositive neurons in the trigeminal ganglion. (A)** Percentage of CLR-immunopositive neurons in untreated animals (Control, n = 2), saline- (n = 6) and GTN-pretreated animals (n = 6). **(B)** CLR-immunopositive neurons specified for the ophthalmic (V1), maxillary (V2) and mandibular (V3) division of trigeminal ganglia in saline and GTN-treated animals. **(C)** Percentage of RAMP1-immunopositive neurons in the trigeminal ganglia of untreated animals (Control, n = 2), saline- (n = 6) and GTN-pretreated animals (n = 6). In the saline- and GTN-treated groups, intensely stained RAMP1-positive neurons are also counted separately. **(D)** RAMP1-positive neurons specified for V1, V2 and V3 in saline- and GTN-treated animals. Error bars represent SD (n=6). * significant difference to saline (Chi-square test of immunopositive and –negative neurons).

In the trigeminal ganglion, neuronal CLR- and RAMP1-immunostaining produced homogenous or faintly granulated immunofluorescence that was distributed over the entire cytoplasm and spared the nucleus (Figure 3C-F). Immunopositive neurons were located in all areas of the trigeminal ganglion. In CLR-immunostained sections of six saline-treated animals, 4618 neurons were counted, 1897 of which were identified as CLR-immunopositive (41% ± 3,8), while among 4659 neurons of six GTN treated animals 1975 neurons were positive (42% ± 4.5). Chi square comparison of immunopositive to immunonegative neurons showed no significant difference between saline and GTN (p = 0.1999; Figure 4A). In RAMP1-immunostained sections of the six saline-treated animals 4967 neurons were counted, 2058 of which were identified as RAMP1-immunopositive (42% ± 4.7), while among 4880 neurons of the six GTN-treated animals 2255 neurons were immunopositive (46% ± 2.3). Chi square comparison of immunopositive to immunonegative neurons showed a significant difference between saline and GTN (p < 0.001; Figure 4C). In the ganglia of the two animals without any pretreatment we counted 780 neurons, 330 of which were identified as CLR-immunopositive (43% ± 0.7) and 1272 neurons, 518 of which were identified as RAMP1-immunopositive (41% ± 2.1). These proportions of immunopositive neuron were not different to the respective proportions in the saline treated animals (Figure 4A and C).

Some neurons regarded to be immunopositive for RAMP1 showed weaker immunofluorescence, while other neurons showed a very intense staining (Figure 3F). Therefore the very intense RAMP1 immunopositive neurons were separately analysed in an additional counting. In the six saline-treated animals we counted 4897 neurons, 1319 of which were classified as very intense RAMP1-immunopositive neurons (27% ± 8.9). In the six GTN treated animals we counted 4738 neurons, 1417 of which were identified as very intense RAMP1-immunopositive (30% ± 6.0). Chi square comparison of immunopositive to immunonegative neurons showed a significant difference between saline and GTN (p = 0.0012; Figure 4C).

The proportions of immunoreactive neurons for CLR and RAMP1 in the individual trigeminal ganglion divisions, ophthalmic (V1), maxillary (V2) and mandibular (V3) are shown in Table 3 and Figure 4B and D. A significant effect of GTN pretreatment on the percentages of RAMP1-immunopositive neurons was seen in V1 and V2 but not in V3.

**Table 3:** RAMP1- and CLR-immunopositive neurons in the V1, V2, V3 regions of the trigeminal ganglia

**RAMP1**							
	**Saline**			**GTN**			**Difference**
							**Saline-GTN**
	RAMP1/total (n)	%	SD (%)	RAMP1/total (n)	%	SD (%)	p-level
V1	605/1484	41%	± 5.7	714/1523	47%	± 5.6	0.0007
V2	632/1566	40%	± 6.2	762/1553	49%	± 6.5	0.0000
V3	824/1917	43%	± 4.2	779/1804	43%	± 2.3	0.9030
**CLR**							
	**Saline**			**GTN**			**Difference**
							**Saline-GTN**
	CLR/total (n)	%	SD (%)	CLR/total (n)	%	SD (%)	p-level
V1	593/1463	41%	± 6.8	820/1394	41%	± 3.3	0.7266
V2	517/1381	37%	± 6.4	606/1520	40%	± 3.3	0.1793
V3	787/1774	44%	± 7.1	795/1745	46%	± 7.4	0.4759

### Non-neuronal CLR and RAMP1-immunoreactivity

**Figure 5:**
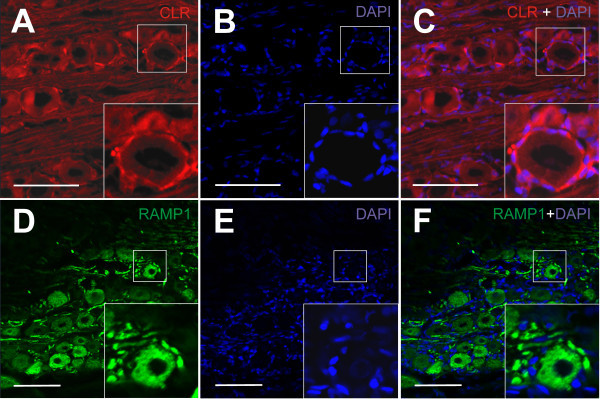
**Immunostaining of non-neuronal cells in the trigeminal ganglion. (A-C)** CLR- (red) and **(D-F)** RAMP1-immunostaining (green) combined with DAPI (blue) nucleus staining. The magnified inset in **(A)** and the DAPI staining **(B)** merged with the CLR staining **(C)** shows an immunonegative neuron with a ring of CLR-immunopositive cells, which most likely represent satellite glial cells. The magnified inset in **(D)** and the DAPI staining **(E)** merged with the RAMP1 staining **(F)** shows a RAMP1-immunopositive neuron with a RAMP1-immunopositive outer hemline, most likely representing satellite glial cells. Scale bars = 100 μm.

DAPI nucleus staining indicated that the trigeminal ganglion in addition to neurons contains many small cells, presumably satellite and Schwann cells. Neurons were frequently surrounded by CLR- and RAMP1-immunoreactive cell structures, which most likely represented satellite glial cells (Figure 5A-F). They were comparable to similar CLR- and RAMP1-immunopositive satellite cells described in previous publications [19,20].

### Double CLR- and RAMP1-immunopositive neurons

Co-localised neurons were identified by CLR-immunopositive staining in the red channel (Alexa-555) and RAMP1-immunopositive staining in the green channel (Alexa-488) (see Figure 3G-I). In the merged confocal images the double-positive neurons appeared either homogeneously or partly yellow, with some variation of intensity.

In ganglion sections from the six saline-treated animals, 2532 neurons were counted, 1057 of which were identified as both CLR and RAMP1 immunopositive (42% ± 7.2). Among 2357 neurons of six GTN-treated animals 933 neurons were positive for both receptor components (40% ± 12.4). Chi square comparison of immunopositive to immunonegative neurons showed no significant difference between saline and GTN (p = 0.1243). Independent from treatment CLR- and RAMP1-immunoreactivity was often co-localized in trigeminal neurons; some neurons expressed only RAMP1, while CLR alone was rarely found.

### Soluble guanylate cyclase-immunoreactive neurons

**Figure 6:**
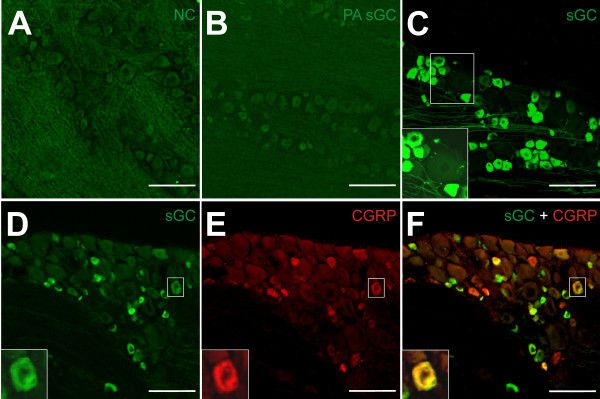
**sGC and CGRP immunostaining of trigeminal ganglia. (A)** Negative control (NC) omitting the primary sGC antibody to control the specificity of the immunostaining with Alexa 488. Neurons show no specific staining. **(B)** Preabsorption control (sGC-peptide: sGC-primary antibody 10:1) with no specific staining. **(C)** sGC immunostaining using Alexa 488 as secondary antibody (green). In the magnified inset the brightness is enhanced to show the negative neurons clearer. The image also shows that sGC-immunopositive neurons are smaller than the sGC-immunonegative neurons. **(D – F)** sGC and CGRP double-immunostaining. **(D)** sGC-positive neurons in the green channel, **(E)** CGRP-positive neurons in the red channel and **(F)** both channels merged with co-localised CGRP and sGC in some neurons. The magnified inset in **(D)** – **(F)** shows a double-positive neuron. Scale bars = 100 μm.

Soluble guanylate cyclase-(sGC-)immunopositive neurons were homogenously and mostly completely stained by immunofluorescense but the nucleus was spared (Figure 6C-F). Cells containing this marker were distributed throughout the trigeminal ganglion.

**Figure 7:**
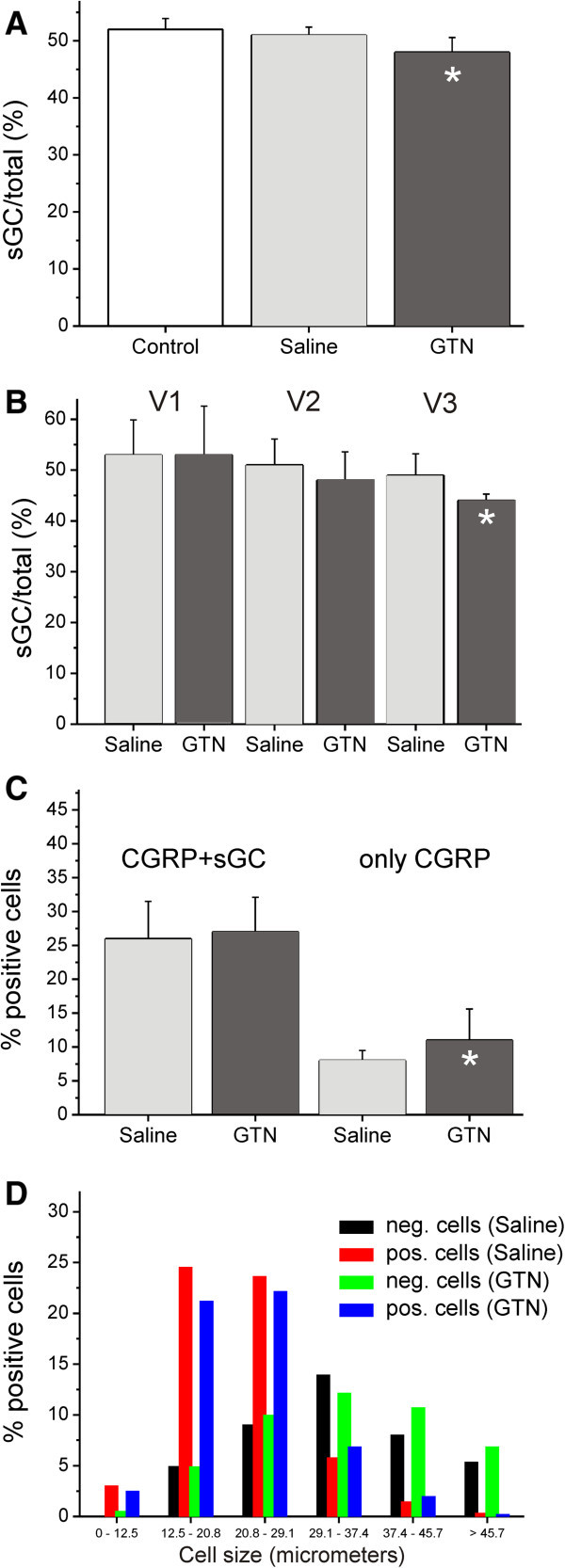
**Quantitative analysis of sGC-immunopositive neurons in the trigeminal ganglion. (A)** Percentage of neurons immunopositive for sGC in the whole trigeminal ganglion and **(B)** in V1, V2 and V3 divisions of the ganglion in control animals (n = 2), saline-treated (n = 6) and GTN-treated (n = 6) animals. **(C)** Double immunopositive neurons for CGRP and sGC in saline- und GTN-treated animals and neurons only immunopositive for CGRP. **(D)** Cell size distribution of sGC-positive and -negative neurons in the trigeminal ganglion in saline- and GTN-treated animals. The sGC-immunopositive neurons are mostly smaller than the sGC-immunonegative neurons in the saline- as well as the GTN-treated group. Error bars represent SD (n=6). * significant difference (Chi-square test of immunopositive and –negative neurons).

In ganglion sections from the six saline-treated animals, 7537 neurons were counted, 3819 of which were identified as sGC-immunopositive (51% ± 1.4), while among 7292 neurons of six GTN treated animals 3523 neurons were found immunopositive (48% ± 2.6). Chi square comparison showed a significant decrease in immunoreactive neurons after GTN compared to saline pretreatment (p = 0.0041). The proportions of immunoreactive neurons for sGC in the trigeminal ganglion divisions V1-V3 are shown in Table 4 and Figure 7A and B, illustrating that the difference between saline and GTN pretreatment was mainly due to the significant decrease in V3. In the ganglia of the two animals, which had not obtained any pretreatment, we counted 2338 neurons, 1213 of which were identified as sGC immunopositive (52% ± 1.9). No difference was seen to saline-treated animals (Figure 7A).

**Table 4:** Proportions of sGC-immunopositive neurons in the V1, V2, V3 regions of the trigeminal ganglia

**sGC**							
	**Saline**			**GTN**			**Difference Saline-GTN**
	sGC/total	%	SD (%)	sGC/total	%	SD (%)	p-level
V1	1253/2376	53%	± 6.9	1210/2272	53%	± 9.6	0.7219
V2	1172/2321	50%	± 5.1	1148/2385	48%	± 5.6	0.1053
V3	1394/2840	49%	± 4.2	1165/2635	44%	± 1.3	0.0003

Most of the sGC-immunopositive neurons were smaller than the sGC-immunonegative neurons, in the saline as well as the GTN-treated group (Figure 7D).

Preabsorption control with the sGC ß1 peptide and negative controls (see Materials and Methods) resulted in no immunoreactivity (see Figure 6B).

### Double CGRP- and sGC-immunopositive neurons

CGRP-immunopositive staining was visualised in the red channel (Alexa-555) and sGC-immunopositive staining in the green channel (Alexa-488). The CGRP immunoreactivity was mostly seen in small neurons homogenously distributed or less frequently clustered around the nucleus. In the merged confocal images the double positive neurons appeared mostly homogenously yellow or clustered more in the center of the cells around the spared nucleus (Figure 6D-F). In six saline-treated animals, 2092 neurons were counted, 173 of which were identified as only CGRP-immunopositive (8% ± 5,9) and 536 as both CGRP- and sGC-immunopositive (27% ± 5.1). Among 2002 neurons of six GTN treated animals 218 neurons were immunopositive only for CGRP (11% ± 4,7) and 522 were immunopositive for CGRP and sGC (26% ± 5.5). Chi square comparison between saline and GTN-treated groups of only CGRP-immunopositive and -immunonegative neurons showed a significant increase after GTN compared to saline pretretment (p = 0.0044), though there was no significant difference in the samples of double CGRP- and sGC-immunopositive neurons (p = 0.7409) (Figure 7C).

## Discussion

The aim of the present study was to investigate immunophenotypic changes of trigeminal neurons after NO-donor treatment. Herein we describe that CGRP receptor components as well as the intracellular NO receptor (sGC) showed changes within distinct territories of the rat trigeminal ganglia. We found that after GTN pretreatment the proportion of trigeminal ganglion neurons immunopositive for the smaller CGRP receptor component, RAMP1, was increased whereas the proportion of neurons immunofluorescent for the large receptor component, CLR, did not change. Furthermore, the ratio of RAMP1-positive neurons was particularly increased in the ophthalmic and maxillary regions of the trigeminal ganglion, which resulted in a significant effect across the whole ganglion. The reason for this differential effect is unknown but may be associated with a higher proportion of ophthalmic (V1) and maxillary (V2) neurons compared to mandibular (V3) neurons innervating intracranial structures.

In agreement with previous data in rat and human trigeminal ganglia [19,20], CLR and RAMP1 were mostly co-localized in trigeminal ganglion neurons, however, some neurons only expressed RAMP1, while CLR was rarely found alone, irrespective of GTN pretreatment. Since CLR and RAMP1 components are required for forming functional CGRP receptors [37] we conclude that the number of functional CGRP receptors may be upregulated after NO donor pretreatment. In previous studies the ratio of CLR-immunopositive trigeminal ganglion neurons was reported to exceed the number of RAMP1-positive neurons [19,20], which was not the case in the present study, possibly due to different fixation procedures or antibodies. Lennerz et al. speculated therefore that RAMP1 is rate-limiting for the formation of functional CGRP receptors, and the selective regulation of RAMP1 could be an economic and time-saving principle to upregulate the number of functional CGRP receptors [20]. The present results do not directly confirm this hypothesis, because the proportion of neurons immunopositive for both receptor components was unchanged, whereas the percentage of neurons selectively immunopositive for RAMP1 increased. It seems plausible that an upregulation of CLR following RAMP1 upregulation in these neurons may occur with some delay, which may have not been captured by our experimental time-scale. It is worth to mention that, consistent with previous data in rat and human trigeminal ganglia [19,20], CLR- and RAMP1-immunoreactivity was very rarely co-localized with CGRP in same neurons, which let us conclude that CGRP may signal from one primary trigeminal afferent unit to another, whereas CGRP autoreception is very unlikely.

Regarding the guanylyl cyclase (sGC) we observed decreased percentages of immunopositive neurons after NO donor pretreatment. Furthermore, sGC-positive neurons were particularly decreased in the mandibular region of the trigeminal ganglion. Cells showing sGC immunoreactivity were small sized, and the size distribution did not change after GTN pretreatment. Approximately one third of the trigeminal ganglion neurons were double-immunopositive for CGRP and sGC. The ratio of neurons double-immunopositive for sGC and CGRP was not changed but the proportion of neurons expressing CGRP but not sGC was increased in the GTN-treated animals. An increase in CGRP-immunoreactive trigeminal ganglion neurons together with an upregulation of neurons immunoreactive to the neuronal NO synthase after GTN pretreatment has previously been described [29].

Collectively these results suggest that systemic treatment with an NO donor like GTN changes the expression pattern of elements of the CGRP and NO signalling. Whereas CGRP-mediated functions may be strengthened by an increase in CGRP and functional CGRP receptors, intracellular NO signalling mediated by sGC may be downregulated, possibly by negative feedback mechanisms.

### Methodical and technical considerations

In this study anti-rat and anti-human antibodies against the CGRP receptor proteins CLR and RAMP1 were tested. For cell counts - the main part of our study - we used the CLR anti-rat and the RAMP1 anti-human-antibodies, because the immunoreactivity was consistent and demonstrated the best contrast over all samples. The experiments from Eftekhari et al. showed full cross-reactivity between rat and human antibodies used in our study [19]. Eftekhari et al. produced and tested the human and rat antibodies with preabsorption and Western blotting [19]. The specificity of the antibodies raised against human and rat CLR and RAMP1 was confirmed in HEK293 cells with stable expression of both the human CGRP receptor components. Preabsorption with the respective blocking peptides resulted in no immnunoreactivity of the antibodies against CLR and RAMP1 in the human or rat trigeminal ganglia [19]. The specificity of CLR and RAMP1 antibodies was also confirmed by Eftekhari et al. with Western blotting [19]. Human and rat CLR antibodies recognized proteins consistent with the molecular weight of CLR and RAMP1. Overall the primary antibodies displayed similar localization and distribution of the staining pattern in human and rat trigeminal ganglia [19].

We detected in saline treated animals 41% CLR-ir positive neurons, 42% RAMP1-ir positive neurons and 27% very intense RAMP1 immunopositive neurons at a section thickness of 20 μm. Lennerz et al. found that 63% of the neurons expressed CLR and 35% of the neurons expressed RAMP1 [20]. The differences to our results could be caused by the use of different primary antibodies or variation in cell counting methods. However, in the previous study using the same antibodies as in the present study, Eftekhari et al. reported that 37% of the neurons were immunopositive for CLR and 36% for RAMP1 at a section thickness of 10–12 μm [19]. The thicker sections of 20 μm in our study could be a reason for the higher numbers of detected RAMP1-immunopositive neurons. In conclusion, the use of identical methods and antibodies seems to be crucical for reliable quantitative comparisons of immunoreactive neurons.

### Distribution and regulation of the soluble guanylyl cyclase

To our knowledge this is the first study that examined the distribution of the sGC in the trigeminal ganglion. Ding et al. showed that sGC is widespread in CNS in rats; both sGC subunits are expressed together almost throughout the whole brain [38]. They suggested that α and β subunits are generally coexpressed in most parts of the brain, forming the enzymatically active heterodimers [38]. In the present study the Santa Cruz antibody against the ß1 subunit of the sGC was used. Based on Ding’s work, we assume that our neurons immunopositive for the ß1 subunit concomitantly express also the α subunit necessary for the functional sGC molecule.

In a study in rat, Western blot analysis and immunhistochemistry after GTN treatment revealed an increase of sGC expression and activity in dural blood vessels, which peaked 30 minutes after the infusion and returned to baseline levels after 60 minutes [28]. The investigators speculated that this phenomenon may be a mechanism to explain the subacute first headache peak occurring within 20–30 minutes after GTN infusion in healthy test persons [39]. However they did not investigate expression and activity of the sGC after a longer post-GTN period, which is of higher interest for the delayed headaches as seen in the clinical trials in migraineurs, who developed migraine-like pain after one to several hours [24].

In our study we observed decreased sGC-immunoreactivity in the trigeminal ganglia six hours after GTN-infusion. This may be an adaptive mechanism reducing the effects of NO. An NO-induced desensitization of sGC has been described in medullary interstitial cells, aortic smooth muscle cells and cardiomyocytes [40-42]. Chronic exposure to NO is known to reduce the sGC activity by decreasing the stability of α1 and ß1 subunit mRNA via transcription- and translations-dependent mechanisms in smooth muscle cells [43]. After intracerebral injection of inflammatory agents in rats decreased expression of sGC at the protein and mRNA level in neural cells occurred [44]. The decrease in sGC-immunoreactivity in our study concerned particularly the mandibular (V3) region of the trigeminal ganglia. The reason for this differential effect is not clear but may be related to a differential upregulation of CGRP receptor components - preferentially in V1 and V2 regions of the ganglion. Provided that V3 contributes less to the intracranial afferent innervation when compared to V1 and V2, the decrease in sGC may serve other functions than meningeal nociception or headache generation.

## Conclusions

In summary we conclude that high NO levels may initiate down-regulation of the guanylyl cyclase. Our results may be transferred to the pathophysiological long-term processes rather than acute or subacute processes which take place in nitrovasodilator-induced and/or spontaneous migraine attacks.

With regard to these adaptive effects of sGC it may be speculated that the delayed excitatory actions of NO donor infusion, i.e. delayed headaches [24] and increase in neuronal activity [30] and c-fos activation [45] in the spinal trigeminal nucleus, is not dependent on a sGC-mediated effect of NO. Alternative mechanisms including actions of NO species like nitroxyl (HNO, NO^-^) via TRP receptor channels have recently been reviewed in detail [46].

## Competing interests

The authors declare that they have no competing interests.

## Authors’ contributions

KS and JIN performed the experiments, analysed the data and drafted the manuscript. JKL and WLN instructed the experimenters and revised the manuscript. KM supervised the experiments and data analysis, drafted and revised the manuscript. All authors read and approved the final manuscript.
